# HMGA2 overexpression plays a critical role in the progression of esophageal squamous carcinoma

**DOI:** 10.18632/oncotarget.8288

**Published:** 2016-03-23

**Authors:** Antonio Palumbo, Nathalia Meireles Da Costa, Francesco Esposito, Marco De Martino, Daniela D'Angelo, Vanessa Paiva Leite de Sousa, Ivanir Martins, Luiz Eurico Nasciutti, Alfredo Fusco, Luis Felipe Ribeiro Pinto

**Affiliations:** ^1^ Programa de Carcinogênese Molecular, Instituto Nacional de Câncer - INCA, Rio de Janeiro, RJ, Brazil; ^2^ Laboratório de Interações Celulares, Instituto de Ciências Biomédicas, Universidade Federal do Rio de Janeiro Prédio de Ciências da Saúde - Cidade Universitária, Ilha do Fundão, Rio de Janeiro, RJ, Brazil; ^3^ Istituto di Endocrinologia e Oncologia Sperimentale - CNR c/o Dipartimento di Medicina Molecolare e Biotecnologie Mediche, Università degli Studi di Napoli “Federico II”, Naples, Italy; ^4^ Divisão de Patologia, Instituto Nacional de Câncer - INCA, Rio de Janeiro, RJ, Brazil

**Keywords:** esophageal cancer, HMGA proteins, cancer progression, diagnostic marker, malignant phenotype reversion

## Abstract

Esophageal Squamous Cell Carcinoma (ESCC) is the most common esophageal tumor worldwide. However, there is still a lack of deeper knowledge about biological alterations involved in ESCC development. High Mobility Group A (HMGA) protein family has been related with poor outcome and malignant cell transformation in several tumor types. In this way, the aim of this study was to analyze the expression of HMGA1 and HMGA2 expression in ESCC and their role in crucial cellular features. We evaluated *HMGA1 and HMGA2* mRNA expression in 52 paired ESCC and normal surrounding tissue samples by qRT-PCR. Here, we show that HMGA2, but not HMGA1, is overexpressed in ESCC samples. This result was further confirmed by the immunohistochemical analysis. Indeed, accordingly to mRNA expression data, HMGA2, but not HMGA1, was overexpressed in approximately 90% of ESCC samples, while it was barely expressed in the respective control. Conversely, HMGA1, but not HMGA2, was overexpressed in esophageal adenocarcinoma samples. Interestingly, HMGA2 abrogation attenuated the malignant phenotype of two ESCC cell lines, suggesting that HMGA2 overexpression is involved in ESCC progression.

## INTRODUCTION

Esophageal cancer (EC) responds for the eighth position in incidence and sixth in mortality among all cancer types, figuring as a highly lethal and poor prognosis tumor [1]. EC is divided into two main histological types, esophageal squamous cell carcinoma (ESCC) and esophageal adenocarcinoma (EAC), which are considerably different regarding the involved etiological factors, affected populations, geographical location and molecular changes involved in the genesis and progression of the disease [2].

Despite the increasing incidence of EAC in developed countries in recent decades [2], ESCC is the most common esophageal tumor and accounts for approximately 80% of all cases, being particularly present in developing countries [3]. Tobacco and alcohol consumption, in addition to a diet deficient in vitamins and minerals, stands out as a major primary risk factor for the development of ESCC [4].

The high lethality displayed by ESCC is mostly due to tumor late stage detection and, consequently, unsuccessful treatment [5]. Even though ESCC ranks as one of the most lethal cancers worldwide, there is still a lack of deeper knowledge about cellular and molecular alterations involved in the genesis and progression of this tumor. Thus, a better understanding of the molecular mechanisms underlying the development and progression of ESCC could be exploited for early diagnosis and/or therapy, promoting a great improvement in disease prognosis.

The High Mobility Group A (HMGA) protein family comprises HMGA1a, 1b, 1c (coded for by the HMGA1 gene through alternative splicing) and HMGA2 (coded by the homonymous gene) proteins which are characterized by their ability to bind to nucleotide sites rich in adenine and thymine [6]. These proteins are unable to directly modulate gene transcription, however, they do so by interacting with the transcriptional machinery and thus altering the transcription factors conformation and chromatin itself, ending up in the stimulation or repression of the expression of several genes [7]. The genes encoding HMGA family members are overexpressed in a wide range of tumors and their overexpression usually correlates with a worse prognosis [8, 9]. HMGA proteins play a role in malignant cell transformation and progression of several tumor types[8–13] and it happens through the modulation of the expression of genes involved in crucial functions, such as cell proliferation and invasion [10].

Therefore, the aim of this study was to analyze the functional expression of HMGA1 and HMGA2 genes and proteins in ESCC tumors and evaluate their role in crucial cellular features *in vitro*. Here, we show that HMGA2, but not HMGA1, is overexpressed in ESCC samples and that the abrogation of its expression in ESCC cell lines attenuates the malignant phenotype.

## RESULTS

### Clinicopathological features

The clinicopathological characteristics of the ESCC patients are presented in Table 1. The median age of patients was 59 years, ranging from 39 to 79 years, male patients represented 76.9% of cases and near 65% of all patients were alcohol consumers and/or smokers. The mean follow up period was of 60 months and the overall survival was of 30.8%. Most of the cases were represented by tumors located at the middle third (57.7%), followed by the upper (26.9%) and the lower third of the esophagus (15.4%). The tumor has been detected in 50% of the patients only at the most advanced stages (II or IV), and poorly or moderately differentiated tumors represented 96.2% of cases.

**Table 1:** Clinicopathological characteristics of the 52 esophageal squamous cell cancer (ESCC) patients comprised in the study. N/A = not informed

Clinicopathological Features	Frequency
**Age**	
Median (years)	59 (39 - 79)
<59	25 (48%)
≥59	27 (52%)
*Total*	52
**Gender**	
Male	40 (76.9%)
Female	12 (23.1%)
*Total*	52
**Smoking**	
Ex smoker	9 (17.3%)
No smoker	8 (15.4%)
Smoker	33 (63.5%)
N/A	2 (3.8%)
*Total*	52
**Alcoholism**	
Ex alcoholic	10 (19.2%)
No alcoholic	8 (15.4%)
Alcoholic	32 (61.5%)
N/A	2 (3.8%)
*Total*	52
**Death**	
No	16 (30.8%)
Yes	36 (69.2%)
*Total*	52
**Tumor Site**	
Lower Third	8 (15.4%)
Medium Third	30 (57.7%)
Upper Third	14 (26.9%)
*Total*	52
**Clinical Stage**	
I	2 (3.8%)
II	15 (28.9%)
III	20 (38.5%)
IV	6 (11.5%)
N/A	9 (17.3%)
*Total*	52
**Tumor Differentiation**	
Low	13 (25%)
Moderately	37 (71.2%)
Well	2 (3.8%)
*Total*	*52*

The overall survival association with all clinicopathological data was performed and a significant association between anatomical site with overall survival (p=0.018) was observed (data not shown).

### HMGA1 is not overexpressed in ESCC tissue

**Figure 1:**
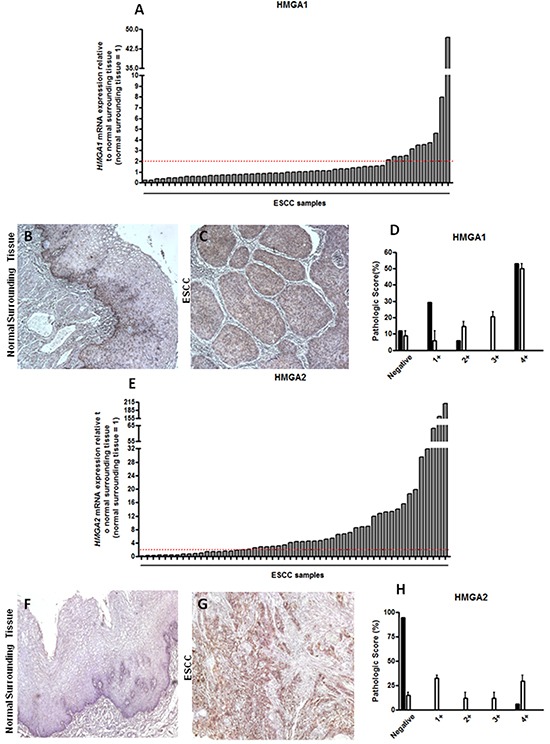
HMGA1 and HMGA2 mRNA and protein expression pattern in esophageal squamous cell carcinomas (ESCC) **A.** qRT-PCR analysis of *HMGA1* mRNA levels in 52 paired ESCC samples. **B.** Representative immunohistochemistry micrographs of histologically normal surrounding mucosa and **C.** ESCC samples stained for HMGA1. **D.** Graphical representation of the 19 ESCC samples HMGA1 staining score. **E.** qRT-PCR analysis of *HMGA2* mRNA levels in 52 paired ESCC samples. **F.** Representative immunohistochemistry micrographs of histologically normal surrounding mucosa **G.** and ESCC samples stained for HMGA2. **H.** Graphical representation of the 19 ESCC samples HMGA2 staining score. mRNA expression values are expressed as relative to those obtained in tumors respective histologically normal surrounding tissue (=1). ESCC samples presenting over 2-fold increase in relative *HMGA1* and *HMGA2* expression were considered upregulated.

In order to analyze *HMGA1* mRNA expression profile, we evaluated its expression in 52 paired ESCC samples by qRT-PCR. This analysis revealed that most of the tumors (78.8%) did not overexpress *HMGA1*, when compared with the expression found in its respective normal surrounding tissue, assuming a fold change cut-off 2 (Figure 1A). In addition, a comparative evaluation of *HMGA1* expression levels distribution between a group of 6 normal esophageal samples (from healthy individuals), the group of the 52 normal surrounding tissues and that of the 52 ESCC was performed and no statistically significant difference was observed between the three groups (data not shown).

Statistical analysis of the association of *HMGA1* gene expression with all clinicopathological data was performed and no significant association between the gene expression and any clinicopathological parameters evaluated in this study was found (Supplementary Table S1).

Next, HMGA1 protein expression was also assessed in 19 paired ESCC samples by immunohistochemistry. Its staining was predominantly nuclear and protein distribution was particularly present in the basal layer of the normal surrounding tissue, as well as in the tumor focus (Figure 1B and 1C). Accordingly to mRNA expression data, HMGA1 protein expression was quite similar among samples, where ESCC and normal surrounding tissue exhibited similar protein expression levels, being it found predominantly in grade 4+ (55% of samples) of pathological score (Figure 1D and Table 2). Moreover, HMGA1 protein expression was absent in only 10% of normal surrounding tissue samples (Figure 1D and Table 2). In this way, these results demonstrate that HMGA1 is not differentially expressed in ESCC when compared with their normal surrounding tissue, neither with healthy esophageal tissue.

**Table 2:** HMGA1 protein expression by immunohistochemistry in 19 paired esophageal squamous cell carcinomas (ESCC) and in 10 esophageal adenocarcinomas (EAC)

HMGA1 Staining Score n (%)					
Histological Type	0	1+	2+	3+	4+
ESCC Normal Surrounding Tissue (n=19)	2(10)	6(30)	1(5)	0	10(55)
ESCC (n=19)	2(10)	2(10)	2(10)	4(20)	9(50)
EAC (n=10)	0	0	0	2(20)	8(80)

### HMGA2 transcript and protein are overexpressed in ESCC samples

**Figure 2:**
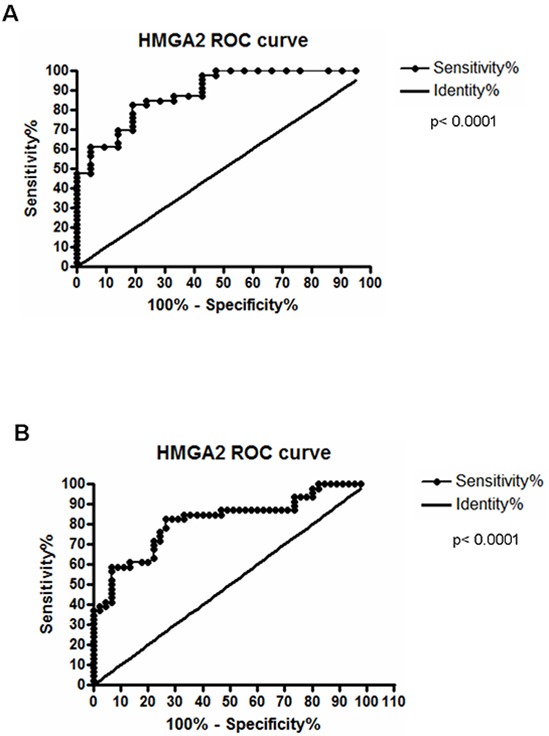
Receiver Operating Characteristc (ROC) analyses **A.** Discrimination of healthy esophageal tissue (n=7) from ESCC samples with 82.61% of sensitivity and 80% of specificity, at a cut-off point of 0.0002329 (area under the curve = 0.8872). **B.** Discrimination of histologically normal surrounding mucosa (n=52) from their paired ESCC samples, being the area under curve (AUC) = 0.8145; Sensitivity = 82.61% and Specificity = 73.33%, at a cut-off point of 0.0002310 (B). Both curves are relative to *HMGA2* mRNA expression.

Subsequently, *HMGA2* mRNA expression was assessed in the same 52 paired ESCC and, at odds with the results achieved for HMGA1, near 64% of the cases presented HMGA2 mRNA levels increased, when compared to their normal surrounding tissue, assuming a fold change cut-off 2 (Figure 1E). Fold change values found in ESCC *HMGA2* expression analysis ranged from 0.13 to 210, with the mean value of 105. Additional analysis comparing the distribution of *HMGA2* expression levels in the 52 ESCC samples with that found in the 52 normal surrounding tissues and in a group of 6 normal esophageal samples (from healthy individuals) showed a significant increase (30-fold increase) in the expression median of ESCC group when compared to the two other groups (Supplementary Figure S1). Similarly to what was found for *HMGA1*, no significant association between *HMGA2* gene expression with all clinicopathological data was observed (Supplementary Table S2). Moreover, no statistically significant correlation between *HMGA2* overexpression and ESCC patients overall survival was detected (Supplementary Figure S2 and Supplementary Table S3). However, in order to evaluate whether HMGA2 possesses diagnostic potential, we performed the Receiver Operating Characteristc (ROC) curve using the expression values from the normal esophageal tissue samples, normal surrounding tissue and ESCC. In this way, ESCC was accurately discriminate from both normal esophageal tissue (p < 0.0001) or normal surrounding tissue (p < 0.0001). Furthermore, sensitivity and specificity were respectively classified with 82.61% and 80.65% (ESCC in comparison with normal esophageal tissue) and with 82.61% and 73.33% (ESCC in comparison with normal surrounding tissue) (Figure 2A and 2B). Therefore, these data could reveal a potential role of HMGA2 as a diagnostic marker in ESCC detection.

Furthermore, as shown in Figure 1F and 1G, HMGA2 protein expression was evaluated by immunohistochemistry. The staining was predominantly nuclear, but occasionally cytoplasmic. The data achieved corroborated the results obtained from mRNA expression analysis. Indeed, HMGA2 protein was detected in approximately 90% of ESCC samples, while in their respective normal surrounding mucosa, the expression was presente in only 5% of samples (Figure 1H and Table 3). Finally, the HMGA2 expression levels showed score grades 2+,3+ and, mainly, 4+ in 50% of ESCC samples (Figure 1H Table 3). Taken together, these results show that the malignant tissues, but not the histological normal esophageal ones, exhibit high levels of HMGA2 mRNA and protein.

**Table 3:** HMGA2 protein expression by immunohistochemistry in 19 paired esophageal squamous cell carcinomas (ESCC) and in 10 esophageal adenocarcinomas (EAC)

HMGA2 Staining Score n (%)					
Histological Type	0	1+	2+	3+	4+
ESCC Normal Surrounding Tissue (n=19)	18(95)	0	0	0	1(5)
ESCC (n=19)	3(16) *	6(32)	2(10)	2(10)	6(32)
EAC (n=10)	1(10)	4(40)	1(10)	2(20)	2(20)

* Statistically significant difference (p ≤ 0.0001) in ESCC HMGA2 expression when compared to their respective histologically normal surrounding mucosa (*t*-Student test).

### HMGA1, but not HMGA2, is overexpressed in EAC

**Figure 3:**
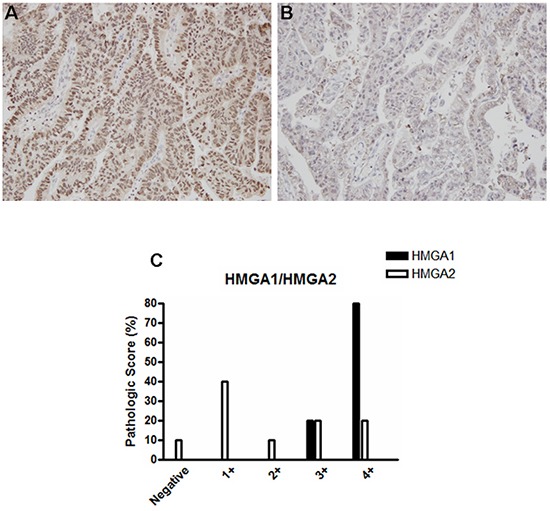
HMGA1 and HMGA2 protein expression pattern in esophageal adenocarcinoma (EAC) samples Representative immunohistochemistry micrographs of EAC samples stained for **A.** HMGA1 and **B.** HMGA2. **C.** Graphical representation of the 10 EAC samples staining score.

Next, since ESCC and EAC represent the two main esophageal tumors histotypes, we investigated whether HMGA1 and HMGA2 expression could differ, according to the histological origin of the esophageal tumors. In this way, we performed HMGA1 and HMGA2 protein expression analysis in 10 EAC samples by immunohistochemistry. In opposite to the results achieved in ESCC samples, HMGA1 was found highly expressed in EAC (Figure 3A and Table 2) tissue while HMGA2 expression was barely expressed in the same samples (Figure 3B and Table 3). Together these results suggest that HMGA1 and HMGA2 proteins are differentially expressed, according to the esophageal tumor histopathological subtype.

### HMGA2 depletion inhibits ESCC cells growth and migration

**Figure 4:**
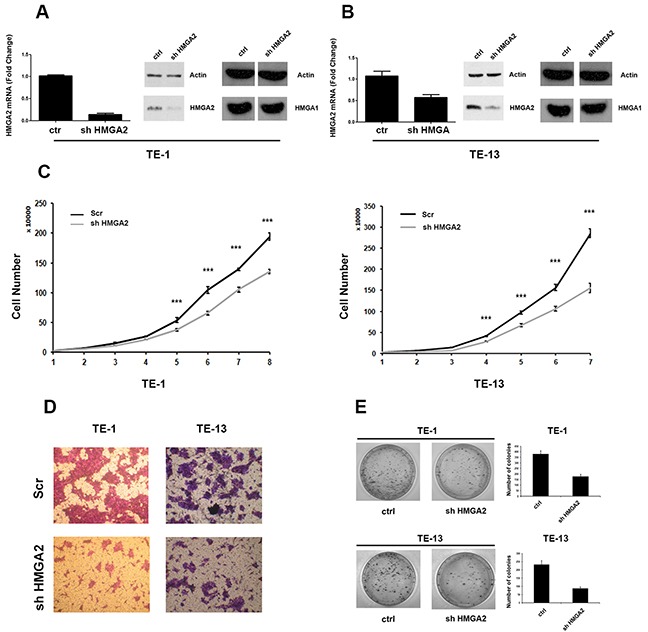
HMGA2 silencing inhibits TE-1 and TE-13 cells growth and migration **A.** qRT-PCR analysis of HMGA2 levels (left panel) and western blot analysis (right panel) of HMGA2 and HMGA1 levels in shHMGA2 transfected TE-1 cells compared to scrambled vector. **B.** qRT-PCR analysis of HMGA2 levels (left panel) and western blot analysis (right panel) of HMGA2 and HMGA1 levels in shHMGA2 transfected TE-13 cells compared to scrambled vector. **C.** TE-1 (left panel) and TE-13 (right panel) cell proliferation in shHMGA2 transfected cells compared to scrambled vector. **D.** Cell migration assay of TE-1 and TE-13 cells transfected with shHMGA2 or with scrambled vector. **E.** Colony formation assay of TE-1 and TE-13 cells transfected with shHMGA2 or with scrambled vector. The number of colonies was quantified by using Quantity One software. *** p < 0,005.

In order to study the role of HMGA2 overexpression in ESCC, vector-mediated RNAi experiments in TE-1 and TE-13 human ESCC cell lines were performed. Then, a shRNA targeting *HMGA2* was used, and the efficiency of knockdown was evaluated by both RT-PCR and Western blot (Figure 4A and 4B). HMGA2 mRNA and protein levels were strongly downregulated by shHMGA2 vector in stable transfected TE-1 and TE-13 cells, with respect to the cells transfected with the scrambled vector (control).

Next, proliferation rates of TE-1 and TE-13 cells knocked-down for HMGA2 were analyzed during 8 consecutive days. As shown in Figure 4C, TE-1 and TE-13 transfected with shHMGA2 grew at a significantly slower rate in comparison with the respective control cells.

Then, a cell migration assay was performed by transfecting TE-1 and TE-13 cells with the shHMGA2 or scrambled control vectors. As shown in Figure 4D, TE-1 and TE-13 shHMGA2 cells displayed a reduced ability to migrate with respect to control cells. Finally, colony-forming efficiency of HMGA2 knocked-down TE-1 and TE-13 cells was assessed by colony-forming assay and it was found that TE-1 and TE-13 sh-HMGA2 cells gave rise to a lower number of colonies compared with control cells (Figure 4E).

Together, these results indicate that HMGA2 expression enhances the malignant phenotype of ESCC cell lines.

## DISCUSSION

Despite the evolution in cancer detection and treatment, ESCC remains as a highly lethal and biologically poor understood tumor, characterized by its late detection and no improvement in therapeutical approaches (chemo and radiotherapy) along the last decades [14, 15]. In this way, the aim of the present work was to investigate the role of HMGA1 and HMGA2 in ESCC by performing *in vitro* and *in vivo* analysis.

Firstly, we observed that *HMGA2*, but not *HMGA1*, mRNA is overexpressed in ESCC samples. Consistently, HMGA2 protein was also upregulated in ESCC specimens, whereas in paired histologically normal surrounding samples its expression was almost absent. Overexpression of HMGA family members, reported in a wide range of tumors, has been already demonstrated to play a critical role in malignant cell transformation and cancer progression [16] and commonly correlates with a poor prognosis [8].

Although our results clearly show that HMGA2 is upregulated in ESCC, no statistically significant correlation between HMGA2 overexpression and ESCC patients overall survival or any other clinicopathological parameter evaluated in this study was observed. Of note, HMGA2 overexpression has been reported as a marker of worse prognosis in several tumors [17–19]. Nevertheless, ESCC represents a more complex landscape since the development of this tumor is not accompanied by clinical symptoms, thus delaying its detection which occurs mostly in advanced stages of the disease, making it difficult to discriminate the malignant development period in which the changes occurred [5]. The ESCC samples collection used in our study reflects this scenario, being near 50% of samples from the most advanced stages and 96% poorly or moderately differentiated. Once HMGA proteins have been demonstrated to play a role in the initial malignant cell transformation and progression [8,9,10,16] the samples analyzed in our study, although representative of ESCC panorama, may not be the best model to study HMGA2 impact on prognosis due to their late disease stage. Thus, the development of models that represent or even mimic the initial steps of ESCC development seems crucial to effectively address the role of HMGA proteins in the genesis and progression of ESCC. On other hand, despite the fact that HMGA2 does not present ESCC prognostic marker value, we observed that *HMGA2* mRNA expression clearly distinguishes ESCC from normal esophageal and surrounding tissue. The importance of the finding that *HMGA2* mRNA expression is capable of discriminating between tumor tissue (ESCC) and the histologically normal surrounding mucosa, as well as between ESCC and healthy esophageal tissues, is highly enhanced by the fact that it has been recently reported that *HMGA2* mRNA can be detected in the plasma of patients affected by other neoplasms [20]. Considering this information, it is possible to test the discriminatory potential of *HMGA2* mRNA expression in individuals with unknown states (“diseased” or “nondiseased”) by using a minimally invasive approach (RNA isolation from plasma). In addition, it is known that the ESCC surrounding mucosa is histologically normal, however, already harboring molecular alterations not found in esophageal healthy tissues [5]. In this way, the identification of a molecular marker differentially expressed in ESCC samples, tumor surrounding mucosa and healthy esophageal tissue represents a potential improvement for ESCC early detection in the near future.

Therefore, to study the consequences of HMGA2 upregulation in ESCC we conducted *in vitro* functional experiments using two ESCC derived cell lines, TE-1 and TE-13. HMGA2 stable knockdown reduced important malignant hallmarks such as proliferation, and migration ability in both cell lines. Moreover, as previously described, It has been reported that HMGA2 overexpression is related with tumor progression in several tissues [19, 21–23] and that it controls the expression of genes in charge of regulating cell proliferation and cell cycle, by mediating different transcriptional mechanisms [8, 9, 10, 24]. For instance, it has been demonstrated that the interaction between HMGA2 and histone deacetylase (HDAC) 1 and 3 inhibits the deacetylation activity of both enzymes, that in turn, favors the acetylation of E2F and Wnt promoter region that are consequently upregulated [11, 12]. In addition, the direct interaction of HMGA2 and ccnb2 promoter region increases the expression of cyclin B2 in pituitary tumors [13].

In accordance with these reports, our results show that HMGA2 knockdown significantly reduced the growth of ESCC cell lines after eight consecutive days in culture, thus suggesting that HMGA2 abrogation could revert the deregulated expression of key genes involved with cell cycle progression and proliferation stimulus.

Moreover, we observed that stable silencing of HMGA2 also reduces the migration and colony formation by ESCC cells TE-1 and TE-13. These observations are indeed interesting since it is known that cellular migration and clustering are directly related with malignant behaviors, such as epithelial mesenchymal transition (EMT) and metastasis process [25, 26]. In fact, it has been shown that HMGA2 plays an essential role in EMT activation [27–30], including an association study with Chinese ethnic group of Kazakh, where a correlation between HMGA2 and Snail expression in ESCC tissue was observed [31]. Furthermore, *in vitro* induced overexpression of miR-154 and Let-7 microRNAs in prostate and esophageal cancer cells abrogates HMGA2 expression and consequently EMT phenotype [32]. In this way, the decrease in migratory activity mediated by HMGA2 abrogation in ESCC cell lines observed in our study could be related with a reversion in EMT phenotype in esophageal malignant cells, nonetheless, further studies are necessary to corroborate this hypothesis. Interestingly, HMGA1 protein expression was highly in EAC samples whereas HMGA2 expression was almost absent in EAC tumors. Despite the fact that ESCC and EAC are both originated in the esophagus, they broadly differ in many aspects, including the etiological factors and the molecular alterations associated with the development of the disease [2]. Therefore, the contrasting expression levels of HMGA1 and HMGA2 in ESCC and EAC could reflect the particular alterations involved in the genesis of squamous cell and adenocarcinomas since a specific-prevalent activation of HMGA2, in some cases in absence of that of HMGA1, has been also observed in other squamous carcinomas such as that of larynx (Palumbo Júnior *et al*. manuscript in preparation and ref [21]), oral cancer [22] and head and neck squamous carcinomas [23].

The genes belonging to HMGA family are expressed during embrionary development while in adult life their expression is completely abrogated in most of the tissues [33]. Some provocative theories propose that cancer results from a re-edition of embrionary mechanisms that, in turn, activate signaling circuits silenced in adult tissues [34–36]. These signaling circuits control important cellular behaviors, which are normally extensively observed during embrionary development, such as proliferation, cell adhesion and migration [37]. In this way, the antagonist HMGA1 and HMGA2 expression pattern observed in esophageal tumors (ESCC and EAC) could result from the activation of specific embrionary-like mechanisms during the distinct development of esophageal squamous cell and adenocarcinoma.

To our knowledge this is the first report that evaluated the functional expression of HMGA2 in ESCC, suggesting that its overexpression could reveal its involvement in ESCC progression, once its abrogation dramatically reduces important malignant hallmarks.

## MATERIALS AND METHODS

### Patients and samples

The gene expression analysis comprised paired biopsies (tumor and histologically normal surrounding tissue, collected at least 5 cm far from the tumor border) obtained from 52 ESCC patients who were submitted to endoscopy, and had confirmed ESCC diagnosis, from 2006 to 2013 at the Brazilian National Cancer Institute (INCA). Epidemiological and clinicopathological data were obtained, respectively, through interviews by using a standardized questionnaire and from patient's medical records. Additional five samples of normal esophageal tissue from healthy individuals who underwent endoscopy due to any reason other than cancer at the Hospital Pedro Ernesto (HUPE / UERJ) were also included in this study. For immunohistochemical analysis, another 22 ESCC samples, and their respective normal surrounding mucosa, were collected from patients who underwent surgery at the Hospital das Clínicas de Porto Alegre (HCPA / UFRGS) and at INCA, from 2006 and 2013, fixed in formalin and embedded in paraffin for further analysis. Tumor tissue and their respective adjacent mucosa were removed respecting 5 cm limit from tumor border. None of the patients comprised in this study had undergone any type of chemotherapy and/or radiotherapy. The use of the human samples was approved by the Ethics Committee of the respective institutions (INCA - 115/10, HUPE / UERJ - 416, HCPA / UFRGS - 02 223). All patients and healthy individuals, who kindly agreed to participate in the study, signed a consent form.

### RNA extraction, reverse transcription and qRT-PCR

Total RNA was extracted from tissue samples and cell lineages using Trizol® reagent, according to the manufacturer's instructions (Invitrogen). All RNA samples were measured by spectrophotometry and 1 μg of RNA was reverse transcribed by using SuperScript™ II Reverse Transcriptase (Invitrogen), following the manufacturer's protocol. *HMGA1 and HMGA2* expression analyses were performed in an Illumina Eco Real-Time PCR System using SYBR Green Master Mix (QiaGen) and oligonucleotides, as follows: *HMGA1* Forward: 5′ AAAAGGACGGCACTGAGAAG 3′, *HMGA1* Reverse: 5′ CTCTTAGGTGTTGGCACTTCG 3′; *HMGA2* Forward: 5′ ccctctaaagcagctcaaaaga 3′, *HMGA2* Reverse: 5′TGGTAGTAGATTGTCCCATTCC 3′; GAPDH Forward: 5′ CAACAGCCTCAAGATCAT CAGCAA 3′, GAPDH Reverse: 5′ AGTGATGGCATGGA CTGTGGTCAT 3′. Each reaction consisted of 5.0 mL of Quantifast SYBR Green PCR Master Mix (Qiagen), 10 pmols of primers and 1 mL of cDNA. The amplification reaction was performed as follows: 5 minutes for DNA pre-denaturation at 95°C, followed by 40 cycles of hybridization and complementary chain synthesis for 5 seconds at 90°C and 10 seconds at 60°C. Each sample was analyzed in triplicate. Relative mRNA levels were calculated using the comparative threshold cycle (CT) with the analyzed gene expression levels normalized by those of *GAPDH* and using the normal surrounding tissue as the reference.

### Immunohistochemistry

Immunohistochemistry (IHC) was performed on 3 μm paraffin sections of 19 ESCC cases and their respective normal surrounding mucosa. For HMGA1 and HMGA2 antigen retrieval, sections were incubated in water bath while submerged in a target buffer solution (DAKO), pH 9.0, for 40 minutes at 98°C. Sections were then incubated with the primaries monoclonal antibodies against HMGA1 (Abcam AB129153, working dilution 1:500) and HMGA2 (Abcam, AB52039, working dilution 1:50), during 12 hours, at least. FFPE anaplastic thyroid carcinoma samples served as positive controls of HMGA1 and HMGA2 staining. As negative control, the primary antibody was replaced by the diluent solution. The detection system used was the NovoLink™ Max Polymer Detection System (Leica Biosystems), following the protocol described by the manufacturer, using diaminobenzidine as substrate - DAKO. Sections were counterstained with Harris’ hematoxylin. The staining score evaluation was performed by three independent pathologists using a light microscope (Nikon, Tokyo, Japan) connected to a digital camera (Coolpix 990; Nikon). For both proteins, scored cases were considered 1+ when positive staining was present in up to 25% of tumor region; 2+ when staining was present between 26% and 50%, 3+ when staining was present between 51% and 75% and 4+ when staining was present between 76% and 100% of tumor region.

### Protein extraction and western blot

Proteins were extracted from the cells by washing them twice in ice-cold PBS and subsequently lysing them by using RIPA-like buffer (250 mMNaCl, 50 mM TRIS-HCl pH 7.4, 0.1% SDS, 2 mM DTT and 0.5% NP-40) containing protease inhibitors (Complete-Mini, Roche). Protein concentration was determined by the Bradford assay (Bio-Rad) using bovine serum albumin as standard and equal amounts of proteins were resolved onto a 12.5% SDS-PAGE, transferred a to nitrocellulose-membrane (Whatman®Protran®) and probed with primary antibodies anti-HMGA1 and anti-HMGA2, as previously described (Esposito et al., 2015). Membranes were then incubated with the horseradish peroxidase-conjugated secondary antibody (1:10,000) and detection was performed with enhanced chemiluminescence (ECL Kit, Amersham).

### Cell lines and transfections

TE-1 and TE-13 cell lines were derived from ESCC and were kindly provided by Dr. Pierre Hainaut (IARC, France). Both lineages were cultured in RPMI medium (Invitrogen) supplemented with 10% fetal bovine serum (Gibco) and 1% of the cocktail penicillin / glutamine / streptomycin (Invitrogen) and maintained at 37°C under 5% CO2. TE-1 and TE-13 cells were stably silenced for *HMGA2* by transfecting 5 μg of DNA from either the shHMGA2 construct plasmid (#HSH019812-LvU6, GeneCopoeia) or the empty backbone vector (#CSHCTR001-LvU6, GeneCopoeia), used as an experimental control, both expressing the puromycin-resistance gene, using Lipofectamine 2000 (Invitrogen), following the manufacturer's protocol. Transfected cells were selected by using 0.5 μg/ml puromycin (Sigma) diluted in the culture medium.

### Proliferation assay

shHMGA2 and backbone vector transfected TE-1 and TE-13 cells were plated in triplicate in a series of 6-cm culture dishes (3 × 10^4^ cells/dish) and counted daily with a cell counter for 8 consecutive days to extrapolate growth curves. The values represent means +/− SEM of the three different experiments.

### Colony assay

TE-1- and TE-13 were transfected with sh*HMGA2* construct plasmid or with the backbone vector (GeneCopoeia). The transfected cells were selected by using 0.5 μg/ml puromycin diluted in the medium used for culture. After 14 days, the cells were fixed and stained with 0.1% crystal violet in 20% methanol and colonies were evaluated.

### Migration assay

Migration rates were evaluated by using transwell culture chambers (Corning Incorporated, Corning, NY). The experiment was performed in 24-well plates containing special inserts made of a polycarbonate filter with 6.5 mm diameter and 8.0 mm pore size. 1×10^5^ cells were previously washed with PBS and then resuspended in RPMI culture medium supplemented with 1% of fetal bovine serum (FBS) before being seeded in the upper chamber. The lower chamber was filled with RPMI supplemented with FBS 10%. The cells were, then, incubated at 37°C for 24 h. After incubation, to block the migration, transwells were removed from wells and stained with 0.05% crystal violet in 25% methanol. Cells that did not migrate were removed from the top of the transwell with a cotton swab, while cells that successfully migrated to the lower chamber remained adherent to the bottom of the membrane.

### Statistical analysis

Frequencies of clinicopathological data and mRNA expression levels of *HMGA1* and *HMGA2* were calculated. For continuous variables, we performed a descriptive analysis of central and dispersion tendencies. To assess the relationship between mRNA expression levels and clinicopathological features, we used the Fisher's exact test. The Kaplan-Meier method was used to evaluate overall survival and disease-free survival, based on a statistically significant confidence interval of 95% and p-value < 0.05. Finally, in order to assess the impact of *HMGA1* and *HMGA2* gene expression profile on overall survival and its statistical significance, Kaplan-Meier Test was performed. Cox regression test was performed with all clinicopathological parameters to adjust the effect of clinical stage and age. Statistical analyzes were performed with GraphPad Prism 5.0 (GraphPad Software Incorporated, USA) and SPSS 17.0. The final values were considered of statistical significance when p < 0.05. To *in vitro* experiments the statistical analysis was performed using the Graph Pad Prism 5.0 following by Anova e Student t test.

## SUPPLEMENTARY FIGURES AND TABLES



## References

[1] Ferlay J, Soerjomataram I, Ervik M, Dikshit R, Eser S, Mathers C, Rebelo M, Parkin DM, Forman D, Bray F (2013). GLOBOCAN 2012 v1.0, Cancer Incidence and Mortality Worldwide: IARC Cancer Base No. 11 [Internet].

[2] Hongo M, Nagasaki Y, Shoji T (2009). Epidemiology of esophageal câncer: orient to ocident. Effects of chronology, geography and ethnicity. Gastroenterology Hepatology.

[3] Vizcaino AP, Moreno V, Lambert R, Parkin DM (2002). Time trends incidence of both major histologic types of esophageal carcinomas in selected countries, 1973-1995. Int J Cancer.

[4] Castellsagué X, Muñoz N, Stefani E, Victora CG, Castelletto R, Rolón PA, Quintana MJ (1999). Independent and joint effects of tobacco smoking and alcohol drinking on the risk of esophageal cancer in men and women. Int J Cancer.

[5] Meireles Da Costa N, Soares Lima SC, Almeida Simão T, Ribeiro Pinto LF (2013). The potential of molecular markers to improve interventions through the natural history of oesophageal squamous cell carcinoma. Biosci Rep.

[6] Johnson KR, Lehn DA, Reeves R (1989). Alternative processing of mRNAs encoding mammalian chromosomal high-mobility-group proteins HMG-I and HMG-Y. Mol and Cell Biol.

[7] Ozturk N, Mehta A, Braun T, Barreto G (2014). HMGA proteins as modulators of chromatin structure during transcriptional activation. Front Cell Dev Bio.

[8] Fusco A, Fedele M (2007). Roles of HMGA proteins in cancer. Nat Can Rev.

[9] Pallante P, Sepe R, Puca F, Fusco A (2015). High Mobility Group A Proteins as Tumor Markers. Frontiers in Medicine.

[10] leynen I, Van de Ven WJM (2008). The HMGA proteins: a myriad of functions (Review). International Journal of Oncology.

[11] Fedele M, Visone R, De Martino I, Troncone G, Palmieri D, Battista S, Fusco A (2006). HMGA2 induces pituitary tumorigenesis by enhancing E2F1 activity. Cancer Cell.

[12] Zong Y, Huang J, Sankarasharma D, Morikawa T, Fukayama M, Epstein JI, Witte ON (2012). Stromal epigenetic dysregulation is sufficient to initiate mouse prostate cancer via paracrine Wnt signaling. PNAS.

[13] De Martino I, Visone R, Wierinckx A, Palmieri D, Ferraro A, Cappabianca P, Fusco A (2009). HMGA proteins up-regulate CCNB2 gene in mouse and human pituitary adenomas. Cancer Research.

[14] Palumbo A, Meireles Da Costa N, Bonamino MH, Ribeiro Pinto LF, Nasciutti LE (2015). Tumor microenvironment gentic instability; A new look at an old neighbor. Mol Can.

[15] Roshandel G, Nourouzi A, Pourshams A, Semnani S, Merat S, Khoshnia M (2013). Endoscopic screening for esophageal squamous cell carcinoma. Arch Of Iran Med.

[16] Fedele M, Fusco A (2010). HMGA and cancer. Biochim Biophys Acta.

[17] Lee J, Ha S, Jung CK, Lee HH (2015). High-mobility-group A2 overexpression provokes a poor prognosis of gastric cancer through the epithelial-mesenchymal transition. Int J Oncol.

[18] Xia YY, Yin L, Tian H, Guo WJ, Jiang N, Jiang XS, Wu J, Chen M, Wu JZ, He X (2015). HMGA2 is associated with epithelial – mesenchymal transition and can predict poor prognosis in nasopharyngeal carcinoma. Onco Targets Ther.

[19] Yang GL, Zhang LH, Bo JJ, Hou KL, Cai X, Chen YY, Li H, Liu DM, Huang YR (2011). Overexpression of HMGA2 in bladder cancer and its association with clinicopathologic features and prognosis HMGA2 as a prognostic marker of bladder cancer. Eur J Surg Oncol.

[20] Galdiero F, Romano A, Pasquinelli R, Pignata S, Greggi S, Vuttariello E, Bello AM, Calise C, Scaffa C, Pisano C, Losito NS, Fusco A, Califano D, Chiappetta G (2015). Detection of high mobility group A2 specific mRNA in the plasma of patients affected by epithelial ovarian cancer. Oncotarget.

[21] Ma LJ, Li W, Zhang X, Huang DH, Zhang H, Xiao JY, Tian YQ (2009). Differential gene expression profiling of laryngeal squamous cell carcinoma by laser capture microdissection and complementary DNA microarrays. Archives of Medical Research.

[22] Sterenczak KA, Eckardt A, Kampmann A, Willenbrock S, Eberle N, Länger F, Kleinschmidt S, Hewicker-Trautwein M, Kreipe H, Nolte I, Escobar HM, Gellrich NC (2014). HMGA1 and HMGA2 expression and comparative analyses of HMGA2, Lin28 and let-7 miRNAs in oral squamous cell carcinoma. BMC Cancer.

[23] Hebert C, Norris K, Scheper M, Nikitakis N, Sauk JJ (2007). High mobility group A2 is a target for miRNA-98 in head and neck squamous cell carcinoma. Molecular Cancer.

[24] Hammond SM, Sharpless NE (2008). HMGA2, MicroRNAs and Stem Cell Aging. Cell.

[25] Chen WL, Kuo KT, Chou TY, Chen CL, Wang CH, Wei YH, Wang LS (2012). The role of cytochrome c oxidase subunit Va in non-small cell lung carcinoma cells: association with migration, invasion and prediction of distant metastasis. BMC Cancer.

[26] Perlikos F, Harrington KJ, Syrigos KN (2013). Key molecular mechanisms in lung cancer invasion and metastasis: A comprehensive review. Crit Rev in Onco/Hemat.

[27] Thuault S, Tan EJ, Peinado H, Cano A, Heldin CH, Moustakas A (2008). HMGA2 and Smads co-regulate SNAIL1 expression during induction of epithelial-to-mesenchymal transition. Journal of Biological Chemistry.

[28] Watanabe S, Ueda Y, Akaboshi S, Hino Y, Sekita Y, Nakao M (2009). HMGA2 maintains oncogenic RAS-induced epithelial-mesenchymal transition in human pancreatic cancer cells. The Amer J of Patho.

[29] Wu J, Liu Z, Shao C, Gong Y, Wei J (2011). HMGA2 overexpression induced ovarian surface epithelial transformation is mediated through regulation of EMT genes. Can Res.

[30] Tan EJ, Thuault S, Caja L, Carletti T, Heldin CH, Moustakas A (2012). Regulation of transcription factor twist expression by the DNA architectural protein high mobility group A2 during epithelial-to-mesenchymal transition. Journal of Biological Chemistry.

[31] Liu Q, Liu T, Zheng S, Gao X, Lu M, Sheyhidin I, Lu X (2014). HMGA2 is down-regulated by microRNA let-7 and associated with epithelial-mesenchymal transition in oesophageal squamous cell carcinomas of Kazakhs. Histopathology.

[32] Zhu C, Li J, Cheng G, Zhou H, Tao L, Cai H, Li P, Cao Q, Ju X, Meng X, Wang M, Zhang Z, Qin C, Hua L, Yin C, Shao P (2013). miR-154 inhibits EMT by targeting HMGA2 in prostate cancer cells. Mol Cell Biochem.

[33] Rogalla P, Drechsler K, Frey G, Hennig Y, Helmke B, Bonk U, Bullerdiek J (1996). HMGI-C expression patterns in human tissues. Implications for the genesis of frequent mesenchymal tumors. Am J of Path.

[34] Soto AM, Sonnenschein C (2005). Emergentism as a default: cancer as a problem of tissue organization. Journal of Biosciences.

[35] Dosch J, Lee CJ, Simeone DM (2001). Stem Cells cancer, and cancer stem cells. Nature.

[36] Hendrix MJ, Seftor EA, Seftor RE, Kasemeier-Kulesa J, Kulesa PM, Postovit LM (2007). Reprogramming metastatic tumour cells with embryonic microenvironments. Nat Rev Cancer.

[37] Bizzarri M, Cucina A, Biava PM, Proietti S, D'Anselmi F, Dinicola S, Pasqualato A, Lisi E (2011). Embryonic morphogenetic field induces phenotypic reversion in cancer cells. Review article. Curr Pharm Biotechnol.

