# Supplementary material for: HMGA2 overexpression plays a critical role in the progression of esophageal squamous carcinoma
Source: Oncotarget. 2016 Mar 23;7(18):25872–84. doi: 10.18632/oncotarget.8288 (PMC5041951; doi:10.18632/oncotarget.8288)
Supplement: Supplementary file 1 [file oncotarget-07-25872-s001.pdf]

SUPPLEMENTARY FIGURES AND TABLES

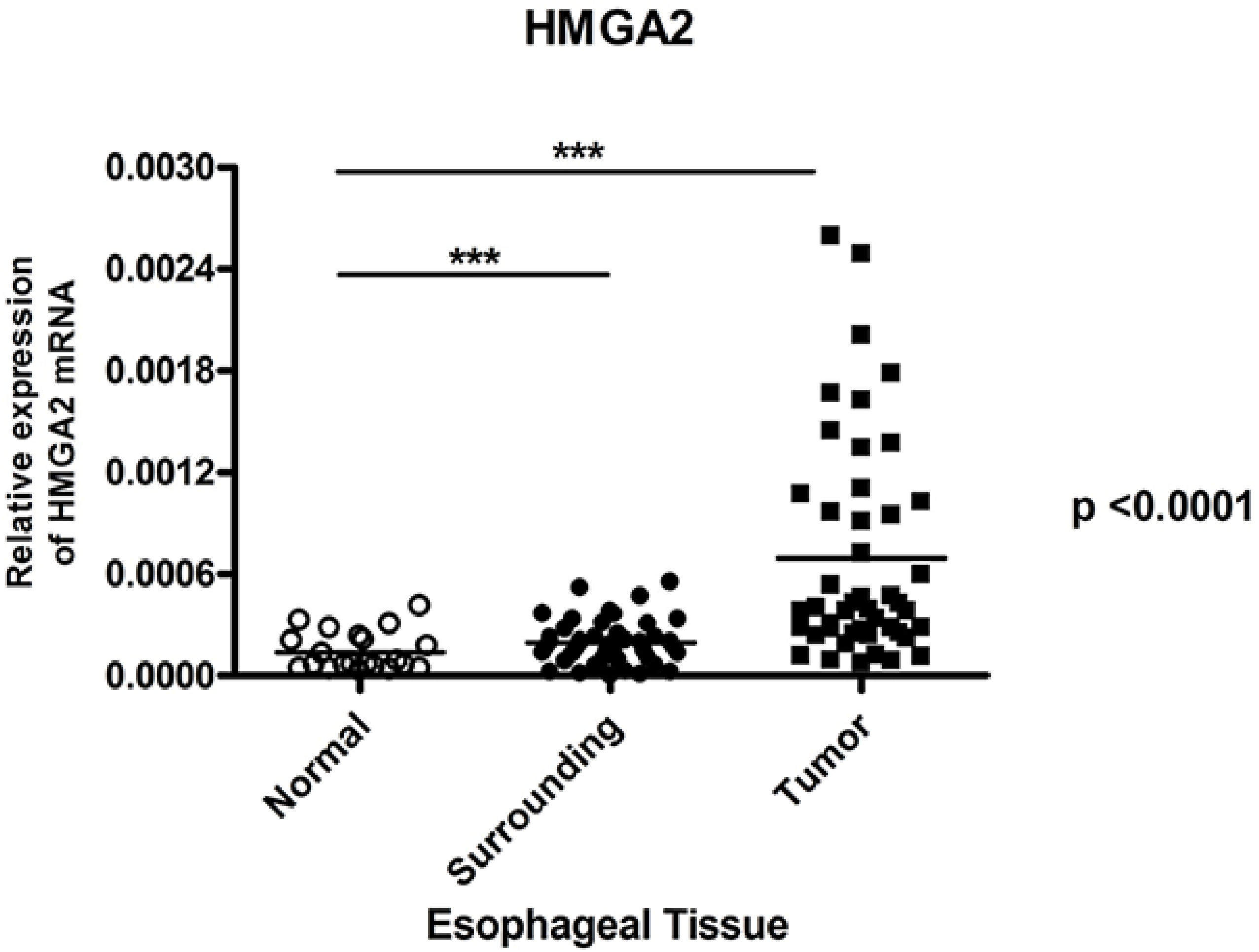

Supplementary Figure S1: *HMGA2* mRNA expression in healthy esophageal, histologically normal surrounding and ESCC tissues. qRT-PCR evaluation of *HMGA2* mRNA levels distribution in the groups of healthy (n=7), histologically normal surrounding (n=52) and their paired ESCC (n=52) tissues. *HMGA2* mRNA levels were normalized by those of *GAPDH*, used as the housekeeping gene.

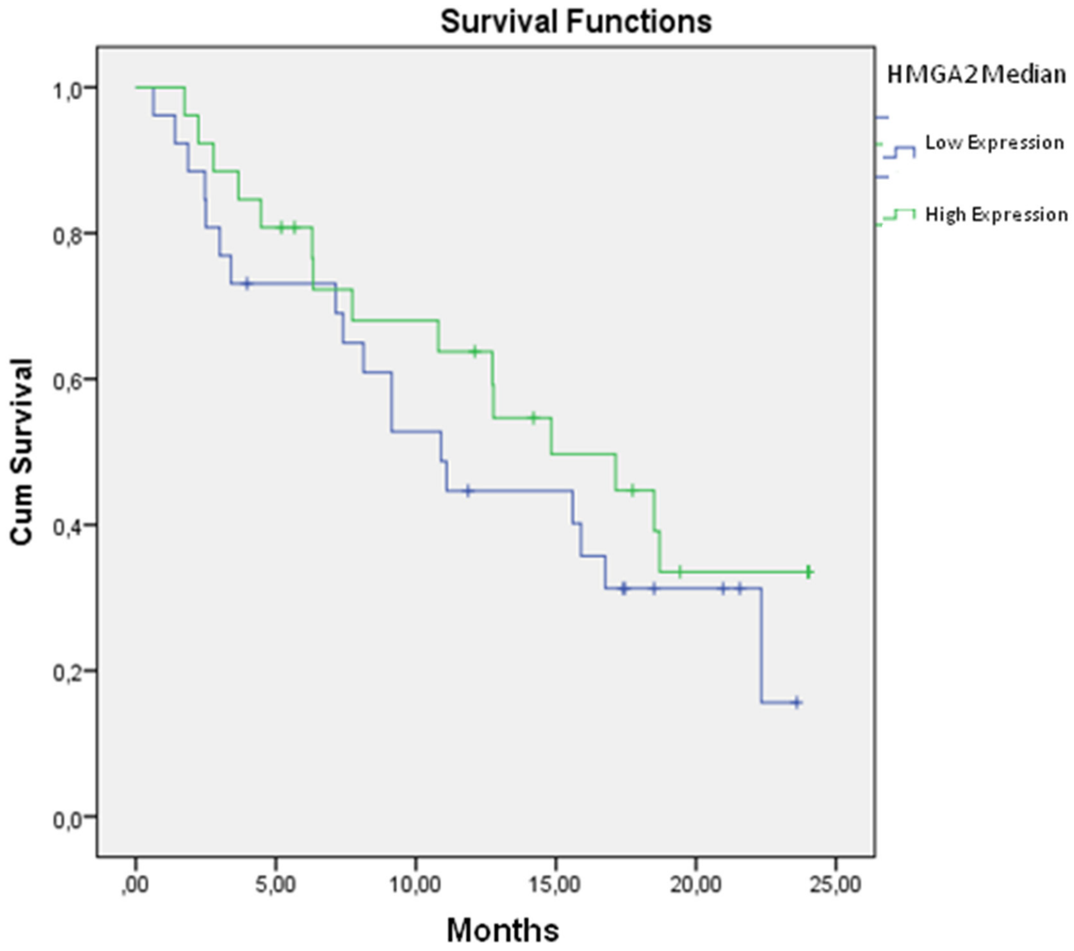

Supplementary Figure S2: Impact of *HMGA2* mRNA expression on esophageal squamous cell carcinomas (ESCC) patients survival. Overall survival analysis of the 52 ESCC patients analyzed in this study. Patients were divided into *HMGA2* low expression group and *HMGA2* high expression group, following the median average value of *HMGA2* mRNA expression observed. Low HMGA2 expression group is represented by the blue line and high HMGA2 expression group is represented by the green line. The Kaplan-Meier method was used to evaluate overall survival, based on a statistically significant confidence interval of 95% and p-value < 0.05.

Supplementary Table S1: Correlations between *HMGA1* gene expression and baseline characteristics of esophageal squamous cell carcinoma (ESCC) patients

	HMGA1		
**Characteristics**	**≤ 0.1696950**	**> 0.1696950**	***p* value**
**Staging TNM**			
I+II	10 (62.5%)	6 (37.5%)	0.3332
III+IV	10 (41.7%)	14 (58.3%)	
**Histological Grade**			
G1 +G2	20 (51.3%)	19 (48.7%)	1.0000
G3	6 (46.2%)	7 (53.8%)	
**Tobacco Consumption**			
Current Smoker	21 (50.0%)	21 (50.0%)	0.7041
Never Smoke	5 (62.5%)	3 (37.5%)	
**Alcohol Consumption**			
Current Drinker	21 (50.0%)	21 (50.0%)	0.7041
Never Drink	5 (62.5%)	3 (37.5%)	
**Third**			
Upper	11 (78.6%)	3(21.4%)	
Middle	11 (36.7%)	19 (63.3%)	**0.0350**
Lower	4 (50.0%)	4 (50.0%)	

Supplementary Table S2: Correlations between *HMGA2* gene expression and baseline characteristics of esophageal squamous cell carcinoma (ESCC) patients

	HMGA2		
**Characteristics**	**≤ 0.00044419**	**> 0.00044419**	***p* value**
**Staging TNM**			
I+II	7 (43.7%)	9 (56.3%)	0.7553
III+IV	12 (50.0%)	12 (50.0%)	
**Histological Grade**			
G1 +G2	18 (46.2%)	21 (53.8%)	0.7523
G3	7 (56.8%)	6 (46.2%)	
**Tobacco Consumption**			
Current Smoker	21 (50.0%)	21 (50.0%)	1.0000
Never Smoke	4 (50.0%)	4 (50.0%)	
**Alcohol Consumption**			
Current Drinker	20 (45.5%)	22 (54.5%)	0.7019
Never Drink	5 (62.5%)	3 (37.5%)	
**Third**			
Upper	8 (57.1%)	6 (42.9%)	
Middle	13 (43.3%)	17 (56.7%)	0.6896
Lower	4 (50.0%)	4 (50.0%)	

Supplementary Table S3: Univariate analysis of *HMGA2* mRNA expression impact on ESCC patients survival

Gene	Tumor Expression	Survival Time				P value
		Median (months)	Pattern Deviation	Confidence Interval 95%		
HMGA-2	Low	12,477	1,660	9,224	15,730	0,398
	High	14,475	1,666	11,509	18,041	
